# The phenomenology of motivation

**DOI:** 10.1192/j.eurpsy.2022.1816

**Published:** 2022-09-01

**Authors:** R. André, J. Romão, F. Azevedo, M. Gonçalves, C. Sereijo, R. Saraiva, M. Croca, M. Abreu

**Affiliations:** 1 Centro Hospitalar Universitário Lisboa Norte, Psychiatry, Lisboa, Portugal; 2 Centro Hospitalar de Lisboa Ocidental, Psychiatry, oeiras, Portugal

**Keywords:** motivation, phenomenology, Psychopathology

## Abstract

**Introduction:**

The concept of motivation pervades our professional and personal lives. Motivation is almost impossible to be observed directly, it is a construct for the interpretation of a behaviour that “calls the attention”.

**Objectives:**

This work reviews the current available data on the phenomenological description of motivation and the abnormalities of motivation.

**Methods:**

Non-systematic review of the literature with selection of scientific articles published in the past 10 years; by searching Pubmed and Medscape databases using the combination of MeSH descriptors. The following MeSH terms were used: “motivation”, “psychopathology”, “phenomenology”.

**Results:**

Abnormalities in motivation may involve diminution or exacerbation. Anhedonia is the absence of pleasure in relation to usually pleasurable activities, it occurs in depression and schizophrenia where the pleasurable intrinsic motivation that acts as incentive for behaviour may be lost. In mania it may be increased so that mundane activities become unduly fascinating and rewarding.

**Conclusions:**

Countless theories have been proposed to explain human motivation but each sheds light on specific aspects of motivation, neglecting others. This diversity creates confusion because most theories have areas of conceptual overlap and disagreement. To facilitate the development of studies, an agreement should be achieved on an operational definition of motivation.

**Disclosure:**

No significant relationships.

